# Emergence of Plasmid-Mediated Fosfomycin-Resistance Genes among *Escherichia coli* Isolates, France

**DOI:** 10.3201/eid2309.170560

**Published:** 2017-09

**Authors:** Yahia Benzerara, Salah Gallah, Baptiste Hommeril, Nathalie Genel, Dominique Decré, Martin Rottman, Guillaume Arlet

**Affiliations:** Assistance Publique des Hôpitaux de Paris Hôpitaux Universitaires Est Parisiens Paris, France (Y. Benzerara, S. Gallah, B. Hommeril, D. Decré, G. Arlet);; Université Pierre et Marie Curie, Sorbonne Université, Paris (N. Genel, D. Decré, G. Arlet);; Assistance Publique des Hôpitaux de Paris Hôpitaux Universitaires Paris Ile de France Ouest, Hôpital Raymond Poincaré, Garches, France (M. Rottman);; Université de Versailles Saint-Quentin-en-Yvelines, St-Quentin en Yvelines, France (M. Rottman)

**Keywords:** fosfomycin, acquired resistance, fosA, phosphonoformiate, Escherichia coli, extended spectrum β-lactamase, ESBL, bacteria, France, foodborne infections, food safety, antimicrobial resistance, enteric infectious

## Abstract

FosA, a glutathione *S*-transferase that inactivates fosfomycin, has been reported as the cause of enzymatic resistance to fosfomycin. We show that multiple lineages of FosA-producing extended spectrum β-lactamase *Escherichia coli* have circulated in France since 2012, potentially reducing the efficacy of fosfomycin in treating infections with antimicrobial drug–resistant gram-negative bacilli.

Fosfomycin is a broad-spectrum bactericidal antibiotic commonly used in Europe as a first-line oral agent for uncomplicated urinary tract infection (*1*). In France, it is the only first-line antimicrobial drug recommended for treatment of cystitis (97% susceptibility) and is used in 20%–30% of such treatments (*2*). However, it is receiving renewed worldwide attention as one of the most active agents for sparing carbapenems in extended spectrum β-lactamase (ESBL)–producing isolates and for treatment of carbapeneme-resistant *Enterobacteriaceae* (CRE) in combination with colistin (*3*). In France, intravenous fosfomycin (3–4 g 4×/d) is used in combination with other drugs for the treatment of multidrug-resistant infections. 

The evaluation of fosfomycin susceptibility in clinical strains is widely performed, but the molecular bases are rarely documented. Fosfomycin inhibits the initial step in peptidoglycan synthesis by irreversibly blocking MurA in both gram-positive and -negative bacteria. It is imported through the inner membrane through the glycerol-3-phosphate (G3P) transporter GlpT and the glucose-6-phosphate (G6P) transporter UhpT. Decreased expression or mutations in *glpT* or *uhpT* genes are the most frequent events leading to lowered susceptibility, whereas modification of the fosfomycin target MurA seems to be rare in clinical isolates (*4*). Another mechanism is the production of FosA, a glutathione *S*-transferase that inactivates fosfomycin by addition of a glutathione residue. This mechanism is particularly relevant because it is disseminative and frequently associated with ESBL-producing *Escherichia coli*. Since 2006, researchers in several countries in East Asia have described plasmid-mediated *fosA3* and, less frequently, *fosA5* (formerly *fosKp96*), which is mostly associated with CTX-M and co-harbored on a conjugative plasmid. Some studies have focused on human clinical strains in China (*5*), South Korea (*6*), or Japan (*7*), and others have addressed veterinary strains isolated throughout China from pets (*8*), livestock (*9*), or animal fodder (*10*). In 2016, Portugal reported the first imported case of a travel-related infection in Europe with an *E. coli* strain co-expressing *fosA3* and CTX-M-15 (*11*). The possible dissemination of this gene is worrisome because *fosA3* is generally surrounded by the IS*26* insertion sequence on a composite transposon borne by the IncFII conjugative plasmid, which is known to be a dissemination vector of resistance genes worldwide. Here we report the prevalence and mechanisms of fosfomycin resistance among clinical human *E. coli* strains isolated in Paris, France.

## The Study

We investigated the occurrence and molecular features of all fosfomycin-resistant *E. coli* isolated from hospitalized patients during a 12-month period (August 2014–July 2015). We performed bacterial identification by using VITEK 2 (BioMérieux, Marcy l’Etoile, France) and tested antibiotic susceptibility by using the disk diffusion method in accordance with 2016 Comité de l’Antibiogramme de la Société Française de Microbiologie/European Committee on Antimicrobial Susceptibility Testing guidelines (http://www.sfm-microbiologie.org/UserFiles/files/casfm/CASFM2016_V1_0_FEVRIER.pdf). We screened for fosfomycin resistance by using a 200-µg disk with a diameter cutoff of <13 mm. We determined MIC by using the Etest method with Muller-Hinton agar containing 25 mg/L G6P. 

Among 1,354 *E. coli* isolates tested, 12 (0.9%) showed confirmed resistance (MIC >128 mg/L). We explored the mechanism of fosfomycin resistance by growing these isolates for 48 hours at 35°C in M9 minimal medium agar supplemented with either G3P or G6P at 0.2% as a sole carbon source. Lack of growth showed impaired fosfomycin transport (*12*). Of the 12 isolates, 7 were double auxotrophic mutants with G6P and G3P (mean MIC 384 mg/L), 3 were auxotrophic only for G3P (mean MIC 597 mg/L), and 2 were capable of using both substrates and exhibited high-level resistance (MIC >1,024 mg/L). Paradoxically, single and double auxotroph strains had lower mean MICs than the 2 nonauxotroph strains. Because transport deficit could not account for the observed phenotype, we screened by PCR and sequenced genes coding enzymatic glutathione S-transferase variants *fosA*, *fosA2*, *fosA3*, *fosA4*, *fosA5*, and *fosC2* (Table 1). In parallel, we screened inhibition of glutathione *S*-transferase activity by using FosA inhibitor phosphonoformiate (Figure) as described by Nakamura et al. (*12*). Results of these 2 tests were in agreement, with each detecting an ESBL-producing strain with enzymatic activity encoded by the *fosA3* gene (MIC >1,024 mg/L). Neither strain had been previously reported in France.

**Table 1:** Oligonucleotide primers used in our study for detection of plasmid-mediated fosfomycin-resistance genes

Target gene	Primer	Sequence, 5′ → 3′	Temp, C°	Amplicon size, bp	Reference
*fosA*	Fwd	ATCTGTGGGTCTGCCTGTCGT	50	271	(*5*)
Rev	ATGCCCGCATAGGGCTTCT

*fosA3*	Fwd	CCTGGCATTTTATCAGCAGT	55	221	(*5*)
Rev	CGGTTATCTTTCCATACCTCAG

*fosA4*	Fwd	CTGGCGTTTTATCAGCGGTT	60	230	This study
Rev	CTTCGCTGCGGTTGTCTTT

*fosA5*	Fwd	TATTAGCGAAGCCGATTTTGCT	55	177	(*5*)
Rev	CCCCTTATACGGCTGCTCG

*fosC2*	Fwd	TGGAGGCTACTTGGATTTG	50	209	(*8*)
Rev	AGGCTACCGCTATGGATTT

*Fwd, forward; Rev, reverse.

**Figure:**
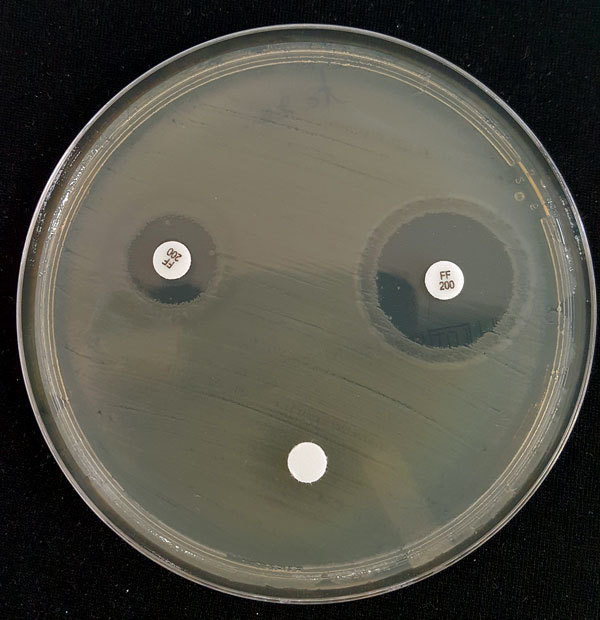
Inhibition of FosA-mediated fosfomycin resistance by phosphonoformiate. A modified Kirby-Bauer disk diffusion susceptibility assay was performed. In brief, a Mueller-Hinton agar plate was streaked with a 0.5 McFarland suspension of the isolate assayed. Three disks were placed on the agar: 200-µg fosfomycin disk (upper left), a 100-µg phosphonoformiate disk (lower center), and a disk with both a 200-µg fosfomycin and 100-µg phosphonoformiate (upper right). The diameter of the growth inhibition zone around each disk was measured after 18–24 h incubation at 35°C (+2°C). FosA-mediated fosfomycin resistance is inhibited by phosphonoformiate and is demonstrated by an increase in the diameter of the growth inhibition zone by >4 mm.

We determined the prevalence of enzymatic resistance to fosfomycin in ESBL-producing strains isolated since 2012 by using the same 2 tests. Surprisingly, among 23 strains resistant to fosfomycin with no epidemiologic link, 7 additional FosA3­-producing and 1 FosA5-producing strains were detected, each with MIC >1,024 mg/L. Overall 83% of fosfomycin-resistant ESBL-producing *E. coli* with MIC >1,024 mg/L were FosA-positive. Auxotrophic tests showed that in addition of FosA production, fosfomycin transport was impaired in 6 strains. Chronologically, 2 strains were isolated in 2012, three in 2013, three in 2014, and two in 2015, meaning that FosA3-producing strains were present in France 6 years after the first isolation in Japan. The FosA5-producing strain was isolated from a clinical sample in France simultaneously with the original description of the strain in China (*13*). The sequencing of *fosA5* showed 99% identity (with 96% coverage) with pHKU1, earlier described as an *fosKP96*-carrying IncN plasmid in *Klebsiella pneumoniae* (*5*).

Our sequencing of CTX-M genes showed that FosA3-producing strains were associated with CTX-M-15 (n = 5), CTX-M-55 (n = 3), and CTX-M-2 (n = 1), whereas the 1 FosA5-producing strain expressed CTX-M-14. We also conducted multilocus sequence typing and plasmid incompatibility group typing (*14*) (Table 2). These results show unambiguously that 8 strains of *E. coli* of different sequence types hosted 5 plasmid types that could be distinguished by their CTX-M variant and plasmid-incompatibility group types.

**Table 2:** Characteristics of clinical fosfomycin-resistant *Escherichia coli* isolates considered in our study*

No. isolates	Year isolated	Origin	CTX-M variant	*fosA* type	Sequence type	Plasmid-carrying *fosA* type
9	2012	Urine	CTX-M-55	A3	ST-559 (ST-10 complex)	FII, I1
12	2012	Urine	CTX-M-55	A3	ST-559 (ST-10 complex)	FII, I1
36	2013	Blood	CTX-M-55	A3	ST-1 (new)	FII
19	2013	Urine	CTX-M-15	A3	ST-2 (new)	FII
34	2013	Urine	CTX-M-2	A3	ST-2015	Nontypeable
24	2013	Urine	CTX-M-15	A3	ST-4508	FII
35	2014	Urine	CTX-M-15	A3	ST-69	FII
39	2014	Joint fluid	CTX-M-15	A3	ST-69	FII
42	2015	Urine	CTX-M-14	A3	ST-457	colE nontypeable
20	2015	Feces	CTX-M-15	A5	ST-3 (new)	N


*All genetic determinants were different except for isolates 9 and 12 (ST-559) and isolates 35 and 39 (ST-69). The yearly number of extended spectrum β-lactamase–producing *E. coli* screened was 1,044 in 2012, 1,142 in 2013, 1,251 in 2014, and 1,381 in 2015. ST, sequence type.

Nine out of 10 isolates yielded transconjugants in *E. coli* C600 (*E. coli* K12 derivative) or transformants in TOP10 (DH10B derivative) *E. coli*. All 9 of these isolates expressed high-level resistance to fosfomycin (MIC *>*1,024 mg/L), confirming that the observed resistance of the parent strain was indeed attributable to the *fosA* gene.

## Conclusions

Although the prevalence of plasmid-mediated *fosA3* genes in human clinical *E. coli* isolates has remained low in France since 2012, these genes are observed across numerous clones, sequence types, and molecular determinants and are always associated with ESBL CTX-M enzymes, suggesting multiple propagation events. Our results are consistent with FosA3-producing clinical strains previously isolated in Asia, which also co-express CTX-M enzymes. However, the CTX-M variant distribution between the strain in France and the strain in Asia are different, with CTX-M-15 having high prevalence in our collection. Medical records examination did not show a history of international travel in our patient population, and such a variety of fosfomycin-resistant *E. coli* lineages probably were not imported or transmitted. The broad use of oral fosfomycin has provided the opportunity to select for FosA producers. With the spread of CTX-M urinary tract infections in the community, the use of fosfomycin is likely to select for CTX-M–FosA co-producers and could lead to an increase of treatment failures with ESBL-producing organisms. Conversely, treatment of ESBL producers with fosfomycin should only be undertaken after testing for susceptibility because these ESBL producers can be linked to the same genetic determinant. Moreover, the indiscriminate use of the oral formulation in the community is jeopardizing the usefulness of this antimicrobial agent. While the world is bracing for an epidemic of infectious diseases bearing plasmid-mediated colistin resistance (*15*), a vast and ubiquitous reservoir for conjugative transmissible resistance to fosfomycin exists and can preclude its efficacy against extremely drug-resistant bacteria if the guidelines for the indiscriminate use of fosfomycin–trometamol are not urgently revised to safeguard this potent and well-tolerated agent. Because antimicrobial treatment of cystitis typically is motivated by concern for patient’s comfort, withholding treatment or the promotion of pivmecillinam as a first-line antimicrobial drug should seriously be considered.

## References

[1] Société de Pathologie Infectieuse de Langue Française. Diagnostic et antibiothérapie des infections urinaires bactériennes communautaires de l’adulte [cited 2017 Jun 21]. http://www.infectiologie.com/UserFiles/File/spilf/recos/infections-urinaires-spilf.pdf

[2] Agence Nationale de Sécurité du Médicaments. Etude d’utilisation de la nitrofurantoïne en France—période mars 2012–février 2015 [cited 2017 Jun 21]. http://ansm.sante.fr/var/ansm_site/storage/original/application/d807c8e39321445201911cf314263f07.pdf

[3] Kaye KS, Gales AC, Dubourg G. Old antibiotics for multidrug-resistant pathogens: from in vitro activity to clinical outcomes. Int J Antimicrob Agents. 2017;49:542–8. 10.1016/j.ijantimicag.2016.11.020 28130072

[4] Castañeda-García A, Blázquez J, Rodríguez-Rojas A. Molecular mechanisms and clinical impact of acquired and intrinsic fosfomycin resistance. Antibiotics (Basel). 2013;2:217–36. 10.3390/antibiotics2020217 27029300 PMC4790336

[5] Ho PL, Chan J, Lo WU, Lai EL, Cheung YY, Lau TC, et al. Prevalence and molecular epidemiology of plasmid-mediated fosfomycin resistance genes among blood and urinary *Escherichia coli* isolates. J Med Microbiol. 2013;62:1707–13. 10.1099/jmm.0.062653-0 23988630

[6] Lee SY, Park YJ, Yu JK, Jung S, Kim Y, Jeong SH, et al. Prevalence of acquired fosfomycin resistance among extended-spectrum β-lactamase-producing *Escherichia coli* and *Klebsiella pneumoniae* clinical isolates in Korea and IS*26*-composite transposon surrounding *fosA3.* J Antimicrob Chemother. 2012;67:2843–7. 10.1093/jac/dks319 22893681

[7] Wachino J, Yamane K, Suzuki S, Kimura K, Arakawa Y. Prevalence of fosfomycin resistance among CTX-M-producing *Escherichia coli* clinical isolates in Japan and identification of novel plasmid-mediated fosfomycin-modifying enzymes. Antimicrob Agents Chemother. 2010;54:3061–4. 10.1128/AAC.01834-09 20404116 PMC2897269

[8] Hou J, Huang X, Deng Y, He L, Yang T, Zeng Z, et al. Dissemination of the fosfomycin resistance gene *fosA3* with CTX-M β-lactamase genes and *rmtB* carried on IncFII plasmids among *Escherichia coli* isolates from pets in China. Antimicrob Agents Chemother. 2012;56:2135–8. 10.1128/AAC.05104-11 22232290 PMC3318358

[9] Chan J, Lo WU, Chow KH, Lai EL, Law PY, Ho PL. Clonal diversity of *Escherichia coli* isolates carrying plasmid-mediated fosfomycin resistance gene *fosA3* from livestock and other animals. Antimicrob Agents Chemother. 2014;58:5638–9. 10.1128/AAC.02700-14 24982077 PMC4135827

[10] Hou J, Yang X, Zeng Z, Lv L, Yang T, Lin D, et al. Detection of the plasmid-encoded fosfomycin resistance gene *fosA3* in *Escherichia coli* of food-animal origin. J Antimicrob Chemother. 2013;68:766–70. 10.1093/jac/dks465 23190765

[11] Mendes AC, Rodrigues C, Pires J, Amorim J, Ramos MH, Novais Â, et al. Importation of fosfomycin resistance *fosA3* gene to Europe. Emerg Infect Dis. 2016;22:346–8. 10.3201/eid2202.151301 26812028 PMC4734505

[12] Nakamura G, Wachino J, Sato N, Kimura K, Yamada K, Jin W, et al. Practical agar-based disk potentiation test for detection of fosfomycin-nonsusceptible *Escherichia coli* clinical isolates producing glutathione *S*-transferases. J Clin Microbiol. 2014;52:3175–9. 10.1128/JCM.01094-14 24951800 PMC4313133

[13] Ma Y, Xu X, Guo Q, Wang P, Wang W, Wang M. Characterization of *fosA5*, a new plasmid-mediated fosfomycin resistance gene in *Escherichia coli.* Lett Appl Microbiol. 2015;60:259–64. 10.1111/lam.12366 25441705

[14] Compain F, Poisson A, Le Hello S, Branger C, Weill FX, Arlet G, et al. Targeting relaxase genes for classification of the predominant plasmids in *Enterobacteriaceae.* Int J Med Microbiol. 2014;304:236–42. 10.1016/j.ijmm.2013.09.009 24342269

[15] Al-Tawfiq JA, Laxminarayan R, Mendelson M. How should we respond to the emergence of plasmid-mediated colistin resistance in humans and animals? Int J Infect Dis. 2017;54:77–84. 10.1016/j.ijid.2016.11.415 27915108

